# Molecular Dynamics Studies on the Structural Stability Prediction of SARS-CoV-2 Variants Including Multiple Mutants

**DOI:** 10.3390/ijms23094956

**Published:** 2022-04-29

**Authors:** Kwang-Eun Choi, Jeong-Min Kim, Jee Eun Rhee, Ae Kyung Park, Eun-Jin Kim, Cheon Kwon Yoo, Nam Sook Kang

**Affiliations:** 1 Graduate School of New Drug Discovery and Development, Chungnam National University, 99 Daehak-ro, Yuseong-gu, Daejeon 34134, Korea; hwendiv@naver.com; 2 Division of Emerging Infectious Diseases, Bureau of Infectious Disease Diagnosis Control, Korea Disease Control and Prevention Agency, 187 Osongsaengmyeong 2-ro, Osong-eup, Heungdeok-gu, Cheongju-si 28159, Korea; jmkim97@korea.kr (J.-M.K.); jerhee001@korea.kr (J.E.R.); parkak1003@korea.kr (A.K.P.); ekim@korea.kr (E.-J.K.); 3 Bureau of Infectious Disease Diagnosis Control, Korea Disease Control and Prevention Agency, 187 Osongsaengmyeong 2-ro, Osong-eup, Heungdeok-gu, Cheongju-si 28159, Korea; ckyoo@korea.kr

**Keywords:** SARS-CoV-2, spike protein, mutant, MD simulation

## Abstract

Severe Acute Respiratory Syndrome Coronavirus 2 (SARS-CoV-2) has caused the Coronavirus Disease (COVID-19) pandemic worldwide. The spike protein in SARS-CoV-2 fuses with and invades cells in the host respiratory system by binding to angiotensin-converting enzyme 2 (ACE2). The spike protein, however, undergoes continuous mutation from a D614G single mutant to an omicron variant, including multiple mutants. In this study, variants, including multiple mutants (double, triple mutants, B.1.620, delta, alpha, delta_E484Q, mu, and omicron) were investigated in patients. The 3D structure of the full-length spike protein was used in conformational analysis depending on the SARS-CoV-2 variants. The structural stability of the variant types was analyzed based on the distance between the receptor-binding domain (RBD) of each chain in the spike protein and the binding free energy between the spike protein and bound ACE2 in the one-, two-, and three-open-complex forms using molecular dynamics (MD) simulation. Omicron variants, the most prevalent in the recent history of the global pandemic, which consist of 32 mutations, showed higher stability in all open-complex forms compared with that of the wild type and other variants. We suggest that the conformational stability of the spike protein is the one of the important determinants for the differences in viral infectivity among variants, including multiple mutants.

## 1. Introduction

Severe Acute Respiratory Syndrome Coronavirus (SARS-CoV-2), an RNA virus, was initially discovered during an outbreak in Wuhan, China and has since become a global pandemic [1]. SARS-CoV-2 invades respiratory organs with symptoms, including cough, followed by fever, myalgia, dyspnea, headache, and death in extreme cases [2]. The number of COVID-19-infected patients has increased continuously, with over 500,000 deaths in the USA (2021) [3]. The four major structural proteins of coronaviruses (CoVs) include spike (S) glycoproteins, envelope (E) proteins, membrane (M) proteins, and nucleocapsid (N) proteins, along with 16 non-structural proteins. The S protein, located in the membrane, plays an important role in viral interactions with the host as it is involved in the first contact with the host cells [4,5]. 

The S protein cleavage by host proteases leads to the production of the S1 subunit at the N-terminus and the S2 subunit at the C-terminus [6]. The biological mechanism for S protein attachment to host cells is caused by the receptor-binding domain (RBD) interaction of the S1 subunit with human angiotensin-converting enzyme 2 (ACE2), including proteases, such as transmembrane serine protease 2, protein cleavage enzyme furin, and extracellular matrix metalloproteinase inducer CD 147 [7]. 

The S1 subunit consists of various domains, namely the N-terminal, RBD, and C-terminal (CT). Depending on the RBD position state, the S protein structure changes into multiple conformational states, such as closed, one-, two-, or full three-open forms [8]. Three RBDs in full open forms can interact with and convert a maximum of three ACE2 molecules from the closed form to the open form. The biological mechanism between ACE2 and RBD has been a research topic for antigenicity under different conditions, such as temperature [9], environment [10], and species [11].

The S protein is continuously variable in protein residues. Multiple amino acid residues of the S protein have mutated evolutionally from a single major D614G mutant to various mutants, including the alpha (from England), beta, delta, mu (from Colombia) [12,13], and omicron variants [14]. In addition, the omicron and delta variants (from India) are the dominant strains globally [15]. However, structural considerations have not been actively researched. Previously, our group reported that single mutants showed higher stability compared with the wild type in all open-complex forms [16]. This study focused on the conformational stability of the various mutants.

Computational methods using released 3D structures have been conducted to investigate the antigenic evolution of viruses [17,18]. Further, these approaches can be used to study the mechanisms of the activity of proteins produced by the virus. Nelson et al. simulated the S protein RBD at the long millisecond scale to study the interaction intensity of ACE2 with the flexible loop region of RBD prior to post-fusion [19]. Deganutti et al. calculated the binding affinity for small molecules to the RBD–ACE2 complex structure [20]. Peng et al. conducted a binding free energy calculation in the system to identify differences in binding affinity of ACE2 for SARS-CoV-2 and SARS-CoV-1 resulting from the 2003 pandemic [21].

The S proteins are frequently variable to avoid the antibodies produced by the host. From April 2020, the single mutant D614G of the S protein exhibited major variations, and D614G became the dominant mutant form of the S protein during the pandemic [22]. Over time, multiple variants, such as alpha, delta, and mu, have been identified. In particular, alpha [23], and delta [24] variants include deletion mutants, whereas omicron variants include both deletion and insertion mutants unlike previous single variants for point mutations. 

Although genomics was actively conducted in the pandemic-struck population, structural research has not been conducted on multiple variants. In this study, we focused on the conformational stability of variants, including multiple mutants, using computational approaches with full-length S protein-ACE2 complex structure in one-, two-, and three-open complexes. Until now, full-length structure-based analysis for S protein-ACE2 complex has not been actively studied unlike only RBD-based structural research. 

In this study, the full-length SARS-CoV-2 S protein–human ACE2 complex structure, including the RBD domain, was selected to analyze the structural stability differences according to variant types, including multiple mutations, such as D614G/E484K double, D614G/E484K/N440K triple mutant, B.1.620, delta lacking E484Q mutation, alpha, delta_E484Q including E484Q mutation, mu, and omicron variants. We expect that this study of the structural stability of SARS-CoV-2 variants, including multiple mutants using the full-length S protein-ACE2 complex, may provide insights explaining the differences in viral infectivity among variant types.

## 2. Results

### 2.1. SARS-CoV-2 S Protein Genome/Protein Analysis in Patients with COVID-19

We identified multiple variants (D614G/E484K double mutant, D614G/E484K/N440K triple mutant, alpha variant, delta variant, mu variant, omicron variant, and B.1.620 lineage) related to the structure of the SARS-CoV-2 S protein using whole-genome sequence analysis from COVID-19 patients in South Korea. E484Q variants were also used, although they have not yet been identified in South Korea.

### 2.2. The Stability Analysis for RBDs in Full-Length Trimeric S Protein Chains

In our previous study, we investigated the structural stability of single mutants. In this study, we focused on multiple variants, and the Protein Data Bank (PDB) data for SARS-CoV-2 S proteins complexed with ACE2 were used. The MD simulation was conducted in the gas phase with a microcanonical (NVE) ensemble as an isolated system with particles (N) and energy (E) in a volume (V) because SARS-CoV-2 infects hosts from the outer air to inner respiratory systems (as does influenza virus [25]), thereby, characterizing the infection process in a variable environment for temperature and pressure. 

We analyzed the stability among chains consisting of trimeric S proteins using the distance and standard deviation (SD) for the distances between two chains (AB and AC, AC and BC, and AC and BC) in each chain for V503 and N501 residues. In order to identify the conformational relationship in each chain of trimeric full-length S proteins, V503 and N501 residues were selected for analysis as in the previous study. V503 residues were involved in the closest distance below 1.0 nm with symmetry among RBDs of chains in closed form (PDB ID 6VXX) of whole trimeric S proteins. 

**Figure 1:**
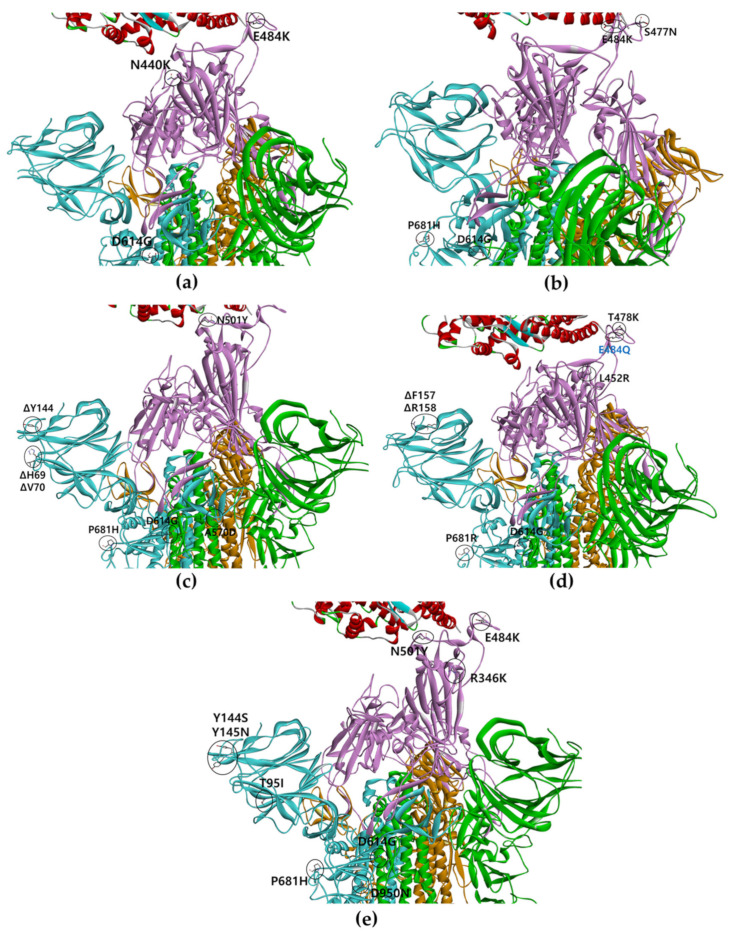
Mutation sites for S protein multiple variants. Trimeric RBDs in each chain are indicated in pink and ACE2 in red. Yellow, blue, and green colors indicate other domains except for RBD in each chain of the S protein. The black circular mark is the mutation site. (**a**) A double/triple mutant, (**b**) B.1.620, (**c**) alpha, (**d**) delta (E484Q mutation lacking in the Korean delta variant), and (**e**) mu variant.

N501 residues involved in the interaction with ACE2 were confirmed as 8.7 nm with the symmetry distance among RBDs of chains in full-open-complex form (PDB ID 7A98) [16]. These analytical methods were used to quantify the stability between the chains of full-length structures considering the V503 residues with three symmetries in the closed form and the N501 residues with three symmetries interacting with the ACE2 interphase in the open form. The mutation sites for each variant are shown in Figure 1. Additionally, the omicron variant with 32 mutation sites (A67V, ΔH69/ΔV70, T95I, G142D/ΔV143-ΔY145, ΔN211/L212I, ins214EPE, G339D, S371L, S373P, S375F, K417N, N440K, G446S, S477N, T478K, E484A, Q493K, G496S, Q498R, N501Y, Y505H, T547K, D614G, H655Y, N679K, P681H, N764K, D796Y, N856K, Q954H, N969K, and L981F) is shown in Figure S1.

**Figure 2:**
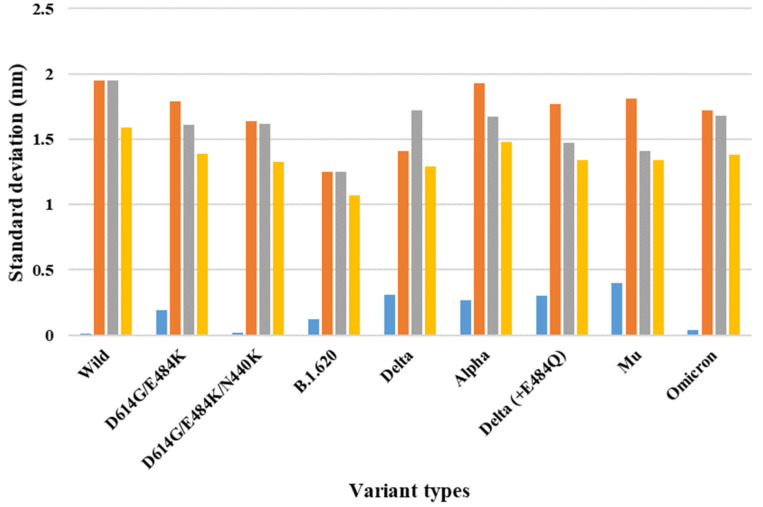
The standard deviation for the distance between V503 residues in each chain for the final MD trajectory in the one-open-complex form (7A94). The *X* axis denotes variant types, and the *Y* axis denotes the standard deviation for distance (nm). Blue indicates the standard deviation (SD) between the AB and BC chains, orange indicates the SD between the AB and AC chains, gray indicates the SD between the AC and BC chains, and yellow indicates the SD among the A, B, and C chains.

**Figure 3:**
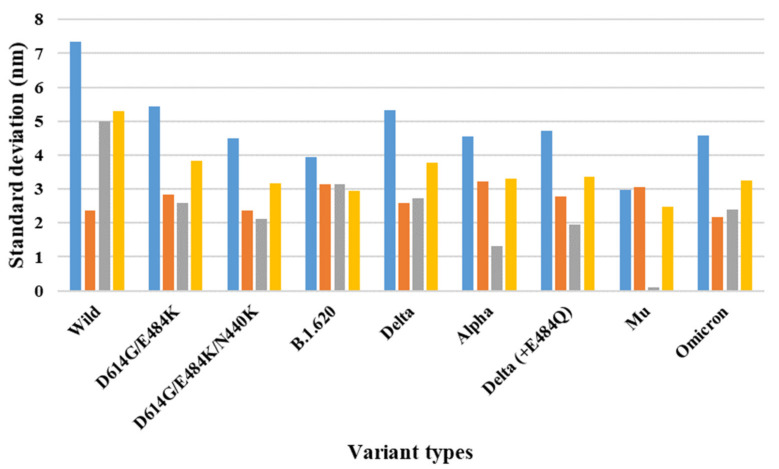
The standard deviation for the distance between V503 residues in each chain for the final MD trajectory in the two-open-complex form (7A97). The *X* axis denotes mutant types, and the *Y* axis denotes the standard deviation for distance (nm). Blue indicates the standard deviation between the AB and BC chains, orange indicates the SD between the AB and AC chains, gray indicates the SD between the AC and BC chains, and yellow indicates the SD among the A, B, and C chains.

**Figure 4:**
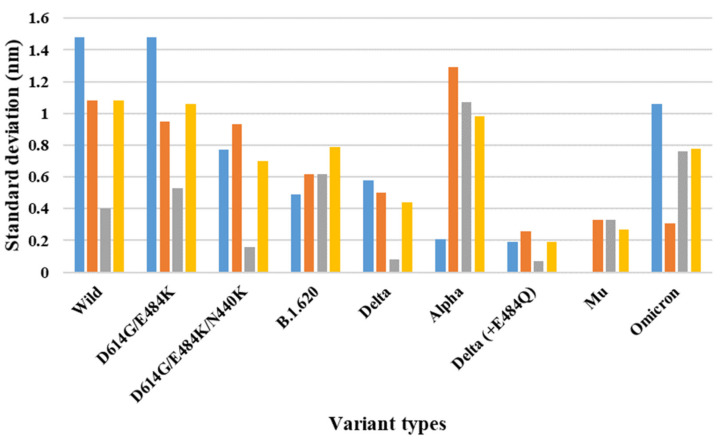
The standard deviation for the distance between V503 residues in each chain for the final MD trajectory in the three-open-complex form (7A98). The *X* axis denotes mutant types, and the *Y* axis denotes the standard deviation for distance (nm). Blue indicates the standard deviation between the AB and BC chains, orange indicates the SD between the AB and AC chains, gray indicates the SD between the AC and BC chains, and yellow indicates the SD among the A, B, and C chains.

Depending on the MD trajectories, the distance and SD of the V503 and N501 residues among the three chains bound to ACE2 were analyzed (Table 1) in all open-complex forms (one-, two-, and three-open). In the one-open-complex form, the SD value for distance calculations showed no differences caused by the different mutation types (<0.5 nm) (Figure 2). In the two-open-complex form, all variants (i.e., double, triple, B.1.620, alpha, delta, delta_E484Q, and omicron) showed commonly lower distance and SD values compared with the wild type. The SD values among the chains were approximately 5 and 4 nm in the wild type and variants, respectively (Figure 3). In the three-open-complex form, all variants had lower SD values compared with the wild type, and delta_E484Q had the lowest SD value among the chains. The mu variant showed also lower distance and SD values (Figure 4).

The raw data for the distance and SD for the distances between two chains (AB and AC, AC and BC, and AC and BC) in each chain for the V503 and N501 residues depending on all MD trajectories are presented in Supplementary Materials. The distance fluctuations between N501 residues showed a pattern similar to that of the V503 residues. In the one-open-complex form, there were no differences caused by the different variants. 

The SD values for the distances among each chain were below 0.5 nm. In the two-open-complex form, the SD values among chains were approximately 4.5 and 4 nm in the wild type and variants (i.e., double, triple, B.1.620, alpha, delta, delta_E484Q, and omicron), respectively. The quantification of the RBDs of the full-length S protein indicated that the variants were more stable than the wild type. From the results for each open-complex form, the delta and mu variants appeared to be more stable compared with the other variants. The summaries of the quantification of V503 residue and N501 residue distances are presented in Table 1 and Table S1, respectively.

### 2.3. Binding Free Energy Analysis on Stability between the S Protein and ACE2

Almost all interaction energies of all RBD–ACE2 complex structures have been studied previously. Our research focused on the energy calculation for all atoms between the full-length S protein and ACE2 using MM/PBSA calculations. The results between the two groups are presented as the van der Waals energy, electrostatic energy, and total energy. Variants consisting of only point mutations and lacking deletion residues were additionally calculated at deleted residue conditions with alpha and delta variant scales. Commonly, in all open-complex forms, the variants were more stable than the wild type. 

**Figure 5:**
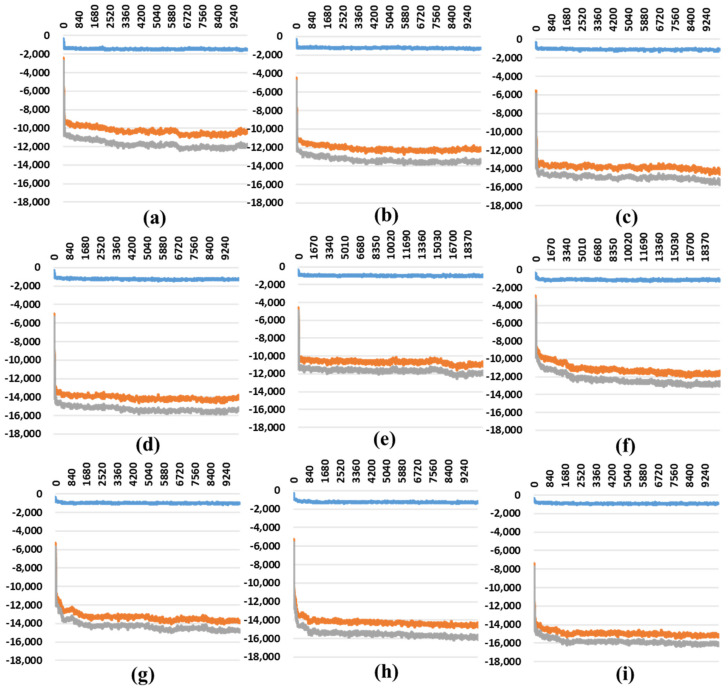
MM/PBSA results depending on the MD trajectories in the one-open-complex form (7A94)**.** (**a**) Wild type and (**b**–**i**) variants. (**b**) double, (**c**) triple, (**d**) B.1.620, (**e**) delta, (**f**) alpha, (**g**) delta_E484Q, (**h**) mu, and (**i**) omicron. The *X* axis denotes the trajectory (ps), and the *Y* axis denotes the MM/PBSA energy value (kJ/mol). Blue indicates the van der Waals energy between the S protein and ACE2, orange indicates electrostatic energy, and gray indicates the total energy.

In the one-open-complex form, the delta and mu variants had lower MM/PBSA values (Figure 5 and Table 2). In the two-open-complex form, the MM/PBSA was calculated between the S protein and ACE2 for the A–D and B–E chains in the two sets. The omicron variant had the lowest stable energy (Figure S8 and Table 3). The dela_E484Q variant remained stable in both steps. In the three-open-complex form, the MM/PBSA was calculated between the S protein and ACE2 for the A–D, B–E, and C–F chains in three sets. The Delta_E484Q variant, except for omicron, showed energetic stability among various variants (Figure S9 and Table 4). 

Other variants, including triple mutants and the B.1.620 variant, showed higher stability but decreased in the delta scale. The omicron variant commonly exhibited the lowest energy in all complex forms.

## 3. Discussion

SARS-CoV-2 is an RNA virus that is currently causing severe pandemic disease. The S protein of SARS-CoV-2 is frequently mutated. The Korea Disease Control and Prevention Agency (KDCA) investigated the S protein variants of SARS-CoV-2 in Korean patients using RT-PCR analysis and identified variants (double, triple, B.1.620, alpha, delta, mu, and omicron) in addition to the previous five major single mutants (D614G, D614A, L455F, F456L, and Q787H) depending on the domain region. In our previous study [16], we showed that the D614G mutant in the CT2 domain has the highest stability among all tested single mutants. In this study, variants, including multiple mutants, were used for the stability analysis. 

Spreading variants, including double, triple, B.1.620, alpha, delta, mu, and omicron variants, were mutated in various domains comprising RBD, CT1, and CT2. The wild type commonly showed lower stability compared with the mutant types through the RBD distance and MM/PBSA values. In particular, the double, triple, B.1.620, delta_E484Q, and mu variants, including both D614G in CT2 and E484K in RBD, showed higher stability compared with the wild type, alpha, and Korean delta variants lacking E484K mutation. In the distance and SD analysis of the distances between two chains (AB and AC, AC and BC, and AC and BC) for the structural stability analysis using V503 and N501 among RBDs, the results for the wild type were similar compared with the variants in one-open-complex form but showed larger SD values in the two- and three-open-complex forms. 

In the binding free energy analysis for stability between the S protein and ACE2 using MM/PBSA, the energy value for the wild type was slightly unstable in the one-open-complex form as expected according to the results from the before-mentioned analysis. The difference in the binding free energies between the wild type and variant types increased significantly in the two- and three-open-complex forms. These results show that the S protein variants, including multiple mutants, have a stronger interaction with ACE2 prior to membrane fusion compared with the wild type. 

The large differences in the analysis for the distance and SD of distances between two chains among the RBDs of the S protein and in the analysis for the binding free energy between the S protein and ACE2 were conspicuous, as these progressed from the one-open form toward the three-open-complex form. These tendencies were clear in variants (double, triple mutants, B.1.620, delta_E484Q, and mu variants), including major mutations, such as D614G and E484K. Therefore, these mutations are expected to play a critical role in the conformational stability for the interaction with ACE2 in the pre-fusion state prior to the metastable state and post-fusion. 

In the metastable state, which requires higher energy than the pre-fusion state, S proteins undergo a rearrangement process to enter membrane fusion between SARS-CoV-2 and the host cell [26]. The conformational stability of the S protein is required for strong interaction with ACE2 in the pre-fusion state prior to the dynamic conformational change to post-fusion [27]. These differences in structural stability in variant types of SARS-CoV-2 were related to the differences in biological function for the interaction with ACE2 in the pre-fusion state from our research results. The alpha and Korean delta variants, with a non-mutation in the E484 residue, showed higher energy than the other variants, including a mutation in the E484 residue, although this showed lower energy than the wild type. 

From these structural perspective, we suggest that the mutation in E484—in addition to D614G as deduced in our previous study [16]—is one of the major determinants in conformational stability. Therefore, although the E484 residue is located in the RBD, and thus its interaction is weak in the interphase between the S protein and ACE2, our study showed that mutation in the E484 residue is related to the conformational stability considering the whole structure using full-length residues in one-, two-, and three-open-complex forms.

In addition to the importance of mutation in the E484 residue, we investigated a relationship between the deletion residues contained in variants, such as delta and alpha and the conformational stability. Deletion residues in the delta variant were reported as avoiding antibody recognition and attack [28]. The binding free energy values in double, triple mutants, B.1.620, and mu variants were decreased in the delta scale (deletion of F157 and R158 residues) but not in the alpha scale (deletion of H69, H70, and Y144). As well as the mutation in E484 residue, deletions in the F157 and R158 residues were slightly involved in conformational stability.

The recently identified omicron variant has not been studied in detail compared to other variants but has been found to be able to avoid the host immune response [29]. The studies by Koleya et al. for the omicron variant using the RBD-ACE2 complex structure resulted in a slightly strong interaction with ACE2 due to showing a lower binding free energy than the wild type [30]. Eileen et al. analyzed the interaction changes of residues in only RBD-ACE2 interphase for wild type, delta, and omicron variants to identify the altered interaction pattern, and two salt-bridge formations in RBD-ACE2 interphase for omicron variants, unlike wild type and delta variants, were found [31]. 

The analysis using only RBD-ACE2, however, considers no conformational stability because the S protein adopts a trimeric whole structure. In our study, we calculated the conformational stability for the omicron variant with 32 mutation sites, including both deletion and insertion in a full-length S protein. The omicron variant showed the largest conformational stability between the S protein and ACE2 in all-open-complex forms based on our results for the binding free energy value using MM/PBSA calculations. 

Therefore, although the recently identified omicron variant has not been actively studied compared with other mutant types, the highest infectivity of the omicron variant among variant types is related to various causes, such as antibody evasion [29], the binding affinity in the RBD-ACE2 interphase [30,31], and the conformational stability considering the whole structure in this study. SARS-CoV-2 variants have evolved into variants, including multiple mutants, such as the omicron variant, in order to enhance the infectivity. As a result of the evolution, the omicron variant appears to have the most stable structure among variant types in connection with increased infectivity. 

It is important to identify and target a large number of mutation sites with conformational stability, such as omicron variants in addition to other variants, including mutations in the D614 and E484 residues for applications in therapeutic development, such as antibody design. Omicron variants, the most prevalent in the recent global pandemic, which consist of 32 mutations, showed higher stability in all open-complex forms compared with the wild type and other variants. We suggest that the conformational stability of the spike protein is an important determinant for the differences in viral infectivity among variants, including multiple mutants.

## 4. Materials and Methods

### 4.1. Ethical Considerations

This study was approved by the Institutional Review Board at the Korea Disease Control and Prevention Agency (2020-03-01-P-A) and is considered to be a public health act to the outbreak. Thus, the board has waived the requirement for written consent as outlined in the Title Laboratory Respondence to COVID-19. All the methods presented in this study were conducted in accordance with the relevant guidelines and regulations.

### 4.2. Genome/Protein Sequence Investigation of Korean Patients with COVID-19

Nasopharyngeal and oropharyngeal swab specimens were collected from symptomatic patients to detect Severe Acute Respiratory Syndrome Coronavirus 2 (SARS-CoV-2) by real-time reverse transcriptase-polymerase chain reaction (RT-PCR). RNA was extracted from the specimens using a Qiagen viral RNA mini kit (Qiagen, Hilden, Germany) according to the manufacturer’s protocol. Next, real-time RT-PCR was performed on the cycle threshold value of the SARS-CoV-2 target gene (ORF 1b and E) [32]. For whole-genome sequencing, the cDNA was amplified using the QIAseq SARS-CoV-2 Primer Panel and QIAseq FX DNA Library UDI Kit (QIAGEN, Hilden, Germany). 

DNA libraries were extracted using the Nextera DNA Flex Library Prep Kit (Illumina, San Diego, CA, USA), and sequencing was performed on the MiSeq instrument using a MiSeq reagent kit V2 (Illumina, San Diego, CA, USA) to obtain an average genome coverage greater than 1000× for all the isolates. The reads were trimmed and mapped to reference genome MN908947.3 using CLC Genomics Workbench version 20.0.3 (CLC Bio, Aarhus, Denmark). Using the reference genome, single-nucleotide variants (SNVs) were called using the BioNumerics version 7.6 SARS-CoV-2 plugin (Applied Maths, Sint-Martens-Latem, Belgium) [33].

### 4.3. Dataset Preparation for SARS-CoV-2 S Protein Structure

The 3D structures of the full-length SARS-CoV-2 S protein complexed with ACE2 were selected prior to the analysis. The PDB structures 7A94, 7A97, and 7A98 were selected as examples of one-, two-, and three-open complexes with ACE2. Variants investigated in Korean patients with COVID-19 were used as double, triple, B.1.620, delta, alpha, mu, and omicron variants; additionally, the delta_E484Q variant identified in other countries was investigated in this study. 3D structures for the variants, including point mutations, were built using the ‘Build and Edit Protein’ module Discovery Studio 2021 (BIOVIA, San Diego, CA, USA). Overall, 24 structures (except for the previous wild type) were built as the inputs to the MD simulation calculation.

### 4.4. Self-Homology Modeling for Deletion Mutant

For the modeling of deletion or insertion mutants, a full-length S protein sequence, including deletion mutants (ΔF157 and ΔR158 for delta variant, and ΔV69, ΔH70, and ΔY144 for alpha variant) and omicron variants (ΔV69, ΔH70, ΔV143, ΔY144, ΔY145, ΔN211, and Ins214EPE) was used as the input. Each open-complex PDB was used as the template structure in SWISS-MODEL [34]. The remaining point mutation residues were built using the ‘Build and Edit Protein’ module in Discovery Studio 2021 (BIOVIA, San Diego, CA, USA).

### 4.5. MD Simulation

All MD simulations for the above 24-input system were performed using the GROMACS 5.1.3 package [35] with the CHARMM27 all-atom force field [36]. The PDB entries 7A94 (one-open-complex form), 7A97 (two-open-complex form), and 7A98 (three-open-complex form) were used as the starting point for the simulations. MD simulations were performed at 298 K in a cubic box with molecules. The system was locally minimized using the steepest descent algorithm with 50,000 steps to ensure that the system had no steric clashes, inappropriate geometry, or structural distortions. MD production was conducted under NVE ensembles in a vacuum phase. 

The Linear Constraint Solver (LINCS) algorithm was used to constrain the bond lengths. A cutoff of 10 Å was used for short-range interactions, long-range electrostatic interactions were treated using the particle-mesh Ewald (PME) summation method [37], and production runs were performed for 10 ns. The integration time-step with leap-frog was set to 1 fs, and the trajectory coordinates and energies were saved at 1 ps intervals. The built-in programs presented as gmx modules of the GROMACS software package were used for the analysis. The re-built 3D structures with self-homology modeling for delta and alpha variants due to deletion mutants conducted more minimization and additional 10 ns MD production in a total of 20 ns.

### 4.6. V503 and N501 Residue Distance Calculation

V503 and N501 residues in RBDs of trimeric S protein chains were selected using the “gmx index” module. The distance calculation between V503 residues and between N501 residues among A–B, A–C, and B–C chains in the S protein were conducted as the minimum distance of residues consisting of atoms in the “gmx mindist” module. Standard deviations (SDs) between V503 residues and between N501 residues in two chains and among three chains, such as A–B and A–C, A–B and B–C, A–C and B–C, and A–B–C, were calculated from the distance values depending on trajectories. The unit for the distance value was represented as nanometers (nm).

### 4.7. Binding Free Energy Analysis Using MM/PBSA Calculation

In order to calculate the binding free energy between the full-length S protein and ACE2 complexes, the molecular mechanics/Poisson–Boltzmann surface area (MM/PBSA) calculation was conducted using the “g_mmpbsa” module [38] in each MD trajectory file. All atom interaction energies between the whole domains of the full-length S protein (not only the RBD) and ACE2 were calculated. 

The structure of 7A94 (one-open-complex form) was used for the MM/PBSA calculation between the A and D chain, and the structure of 7A97 (two-open-complex form) was used for the MM/PBSA calculation between the B and E chain in addition to calculation between the A and D chain. The structure of 7A98 (three-open-complex form) was used for the MM/PBSA calculation between the A–D, B–E, and C–F chains. Due to the long computation time, the MM/PBSA was calculated at 10 ps intervals. 

The van der Waals, electrostatic, and total energies were represented as kJ/mol in the vacuum phase system. All variants in the dataset preparation were used in this binding free energy calculation. In order to correct an effect for deletion residues, such as alpha and delta variants, the binding free energy for the wild type and the variant types consisting of only point mutants, such as double, triple mutants, B.1.620, and mu variants, was also calculated with deletion condition (ΔF157 and ΔR158 for delta variant, and ΔV69, ΔH70, and ΔY144 for alpha variant) in the final trajectory.

## Figures and Tables

**Table 1:** Summary of distance and SD between V503 residues (nm). The values were measured in the final trajectory (10 ns).

PDB	Variant Type	A–B	A–C	B–C	S.D. (A–B) & (A–C)	S.D. (A–B) & (B–C)	S.D. (A–C) & (B–C)	S.D. (A–B–C)
7A94 (one-open-complex form)	Wild	3.00	3.01	0.25	0.01	1.95	1.95	1.59
	D614G/E484K	2.78	2.51	0.24	0.19	1.79	1.61	1.39
	D614G/E484K/N440K	2.78	2.75	0.46	0.02	1.64	1.62	1.33
	B.1.620	2.55	2.72	0.78	0.12	1.25	1.25	1.07
	Delta	2.39	2.83	0.40	0.31	1.41	1.72	1.29
	Alpha	2.96	2.58	0.22	0.27	1.93	1.67	1.48
	Delta (+E484Q)	3.08	2.66	0.58	0.30	1.77	1.47	1.34
	Mu	2.95	2.39	0.40	0.40	1.81	1.41	1.34
	Omicron	3.33	3.28	0.91	0.04	1.72	1.68	1.38
7A97 (two-open-complex form)	Wild	13.96	3.57	10.63	7.35	2.36	5.00	5.31
	D614G/E484K	11.24	3.57	7.23	5.43	2.83	2.59	3.84
	D614G/E484K/N440K	9.52	3.16	6.16	4.49	2.37	2.12	3.18
	B.1.620	9.79	4.23	5.35	3.93	3.14	3.14	2.94
	Delta	11.13	3.59	7.45	5.33	2.60	2.73	3.77
	Alpha	10.65	4.23	6.09	4.55	3.23	1.32	3.31
	Delta (+E484Q)	10.59	3.92	6.66	4.72	2.78	1.94	3.35
	Mu	8.20	4.00	3.86	2.97	3.07	0.10	2.47
	Omicron	9.81	3.34	6.73	4.57	2.18	2.40	3.24
7A98 (three-open-complex form)	Wild	8.23	10.32	9.75	1.48	1.08	0.40	1.08
	D614G/E484K	7.72	9.81	9.06	1.48	0.95	0.53	1.06
	D614G/E484K/N440K	7.63	8.72	8.95	0.77	0.93	0.16	0.70
	B.1.620	8.71	8.02	9.59	0.49	0.62	0.62	0.79
	Delta	9.18	10.00	9.88	0.58	0.50	0.08	0.44
	Alpha	8.80	9.10	10.62	0.21	1.29	1.07	0.98
	Delta (+E484Q)	9.26	8.99	8.89	0.19	0.26	0.07	0.19
	Mu	9.60	9.60	9.14	0.00	0.33	0.33	0.27
	Omicron	10.32	8.81	9.89	1.06	0.31	0.76	0.78

**Table 2:** Summary of the MM/PBSA between the S protein and ACE2 in the one-open-complex form. VdW indicates van der Waals energy, E denotes electrostatic energy, and T is the total energy (kJ/mol). The values were measured in the final trajectory (10 ns).

Variant Type	V	E	T
Wild	−1461.5	−10,311.8	−11,773.3
Wild_deltascale (ΔF157/ΔR158)	−1456.7	−9227.7	−10,684.4
Wild_ alphascale (ΔH69/ΔV70/ΔY144)	−1457.6	−10152.2	−11609.8
D614G/E484K	−1283.4	−12,360.2	−13,643.7
D614G/E484K_deltascale (ΔF157/ΔR158)	−1280.2	−11,380.0	−12,660.2
D614G/E484K_alphascale (ΔH69/ΔV70/ΔY144)	−1283.1	−12,359.2	−13,642.3
D614G/E484K/N440K	−1116.2	−14,581.0	−15,697.2
D614G/E484K/N440K deltascale (ΔF157/ΔR158)	−1114.5	−13,526.4	−14,640.9
D614G/E484K/N440K_alphascale (ΔH69/ΔV70/ΔY144)	−1115.0	−14,570.2	−15,685.2
B.1.620 (S477N/E484K/D614G/P681H)	−1318.0	−13,832.5	−15,150.5
B.1.620_deltascale (ΔF157/ΔR158)	−1316.3	−12,772.9	−14,089.2
B.1.620_alphascale (ΔH69/ΔV70/ΔY144)	−1317.7	−13,821.9	−15,139.6
Delta (ΔF157/ΔR158/L452R/T478K/D614G/P681R)	−968.9	−11,150.0	−12,118.9
Alpha (ΔH69/ΔV70/ΔY144/N501Y/A570D/D614G/P681H)	−1157.5	−11,690.1	−12,847.7
Delta_E484Q (VF157/ΔR158/L452R/T478K/D614G/P681R/E484Q)	−1046.3	−13,738.3	−14,784.6
Mu (T95I/Y144S/Y145N/R346K/E484K/N501Y/D614G/P681H/D950N)	−1290.4	−14,776.8	−16,067.2
Mu_deltascale (ΔF157/ΔR158)	−1280.3	−13,360.1	−14,640.4
Mu_alphascale (ΔH69/ΔV70/ΔY144)	−1290.0	−14,782.2	−16,072.2
Omicron	−921.2	−16,059.5	−16,980.7

**Table 3:** Summary of the MM/PBSA between the S protein and ACE2 in the two-open-complex form. V indicates van der Waals energy, E denotes electrostatic energy, and T is the total energy (kJ/mol). The values were measured in the final trajectory (10 ns).

Variant Type	7A97 AD			7A97 BE			7A97 Total		
	V	E	T	V	E	T	V	E	T
Wild	−1326.4	−13,200.4	−14,526.7	−677.3	−5553.0	−6230.3	−2003.7	−18,753.4	−20,757.0
Wild_deltascale (ΔF157/ΔR158)	−1305.0	−11,646.9	−12,951.9	−677.2	−5195.1	−5872.3	−1982.3	−16,842.0	−18,824.3
Wild_alphascale (ΔH69/ΔV70/ΔY144)	−1311.7	−12,967.2	−14,278.9	−677.2	−5550.9	−6228.1	−1989.0	−18,518.1	−20,507.1
D614G/E484K	−1265.9	−13,766.8	−15,032.8	−725.2	−8535.5	−9260.7	−2003.2	−20,445.4	−22,448.6
D614G/E484K_deltascale (ΔF157/ΔR158)	−1278.0	−12,305.2	−13,583.2	−725.2	−8140.3	−8865.5	−2003.2	−20,445.6	−22,448.8
D614G/E484K_alphascale (ΔH69/ΔV70/ΔY144)	−1250.0	−13,611.7	−14,861.7	−725.2	−8538.6	−9263.8	−1975.3	−22,150.4	−24,125.6
D614G/E484K/N440K	−874.3	−13,307.9	−14,182.2	−626.7	−10,773.4	−11,400.1	−1504.7	−22,321.5	−23,826.2
D614G/E484K/N440K_deltascale (ΔF157/ΔR158)	−878.0	−11,944.6	−12,822.6	−626.7	−10,376.9	−11,003.6	−1504.7	−22,321.5	−23,826.2
D614G/E484K/N440K_alphascale (ΔH69/ΔV70/ΔY144)	−873.4	−13,308.3	−14,181.8	−626.7	−10,774.4	−11,401.1	−1500.1	−24,082.7	−25,582.9
B.1.620 (S477N/E484K/D614G/P681H)	−1696.4	−14,874.1	−16,570.6	−676.0	−10,506.1	−11,182.1	−2368.8	−23,361.0	−25,729.8
B.1.620_deltascale (ΔF157/ΔR158)	−1692.8	−13,415.8	−15,108.6	−676.0	−9945.1	−10,621.1	−2368.8	−23,360.9	−25,729.7
B.1.620_alphascale (ΔH69/ΔV70/ΔY144)	−1647.6	−14,698.5	−16,346.1	−676.0	−10,500.0	−11,176.0	−2323.6	−25,198.5	−27,522.1
Delta (ΔF157/ΔR158/L452R/T478K/D614G/P681R)	−1344.8	−13,867.5	−15,212.3	−534.5	−8634.5	−9169.0	−1879.3	−22,502.0	−24,831.3
Alpha (ΔH69/ΔV70/ΔY144/N501Y/A570D/D614G/P681H)	−1273.8	−11,310.1	−12,583.9	−917.2	−6604.0	−7521.2	−2191.0	−17,914.1	−20,105.1
Delta_E484Q (ΔF157/ΔR158/L452R/T478K/D614G/P681R/E484Q)	−1385.1	−13,658.2	−15,043.3	−676.6	−10,319.8	−10,996.4	−2061.7	−23,978.0	−26,039.7
Mu (T95I/Y144S/Y145N/R346K/E484K/N501Y/D614G/P681H/D950N)	−1084.8	−13,097.6	−14,182.3	−1356.9	−14,208.0	−15,564.9	−2441.7	−27,305.6	−29,747.2
Mu_deltascale (ΔF157/ΔR158)	−1093.6	−11,809.3	−12,902.9	−1356.4	−13,230.0	−14,586.4	−2450.0	−25,039.3	−27,489.3
Mu_alphascale (ΔH69/ΔV70/ΔY144)	−1074.1	−13,035.9	−14,110.0	−1355.7	−14,169.2	−15,524.9	−2429.8	−27,205.1	−29,634.9
Omicron	−1343.9	−16,994.6	−18,338.5	−1215.5	−17,733.8	−18,949.3	−2559.5	−34,728.4	−37,287.8

**Table 4:** Summary of the MM/PBSA between the S protein and ACE2 in the three-open-complex form. V indicates van der Waals energy, E denotes electrostatic energy, and T is the total energy (kJ/mol). The values were measured in the final trajectory (10 ns).

Variant Type	7A98 AD			7A98 BE			7A98 CF			7A98 Total		
	V	E	T	V	E	T	V	E	T	V	E	T
Wild	−535.4	−5278.5	−5813.8	−527.7	−4872.3	−5400.0	−583.0	−5587.4	−6170.4	−1646.1	−15,738.2	−17,384.2
Wild_deltascale(ΔF157/ΔR158)	−535.4	−4883.9	−5419.3	−527.7	−4404.4	−4932.1	−583.0	−5176.7	−5759.7	−1646.0	−14,465.0	−16,111.0
Wild_ alphascale (ΔH69/ΔV70/ΔY144)	−535.4	−5276.2	−5811.6	−527.7	−4873.3	−5401.0	−583.0	−5586.5	−6169.5	−1646.0	−15,736.1	−17,382.1
D614G/E484K	−1236.3	−12,616.4	−13,852.7	−931.4	−13,292.4	−14,223.9	−710.6	−10,229.1	−10,939.7	−2878.3	−36,137.9	−39,016.3
D614G/E484K_deltascale (ΔF157/ΔR158)	−1236.0	−11,740.8	−12,976.9	−931.1	−12,398.8	−13,329.9	−710.3	−9402.2	−10,112.6	−2877.4	−33,541.8	−36,419.4
D614G/E484K_ alphascale (ΔH69/ΔV70/ΔY144)	−1209.9	−12,601.0	−13,810.9	−909.4	−13,278.9	−14,188.3	−717.8	−10,124.4	−10,842.2	−2837.1	−36,004.3	−38,841.4
D614G/E484K/N440K	−1203.1	−12,512.8	−13,716.0	−1351.0	−15,489.1	−16,840.2	−1010.8	−12,006.6	−13,017.4	−3565.0	−40,008.5	−43,573.6
D614G/E484K/N440K_deltascale (ΔF157/ΔR158)	−1202.8	−11,658.0	−12,860.9	−1350.7	−14,612.7	−15,963.4	−1009.5	−11,050.2	−12,059.8	−3563.0	−37,320.9	−40,884.1
D614G/E484K/N440K_alphascale (ΔH69/ΔV70/ΔY144)	−1171.4	−12,430.8	−13,602.2	−1276.9	−15,413.7	−16,690.5	−1000.6	−11,966.5	−12,967.1	−3448.8	−39,811.0	−43,259.8
B.1.620 (S477N/E484K/D614G/P681H)	−866.3	−11,861.2	−12,727.5	−733.9	−10,837.8	−11,571.8	−1275.6	−13,000.2	−14,275.8	−2875.8	−35,699.2	−38,575.1
B.1.620_deltascale (ΔF157/ΔR158)	−866.0	−10,973.5	−11,839.5	−733.7	−10,017.6	−10,751.3	−1275.4	−12,173.6	−13,449.0	−2875.1	−33,164.7	−36,039.8
B.1.620_alphascale (ΔH69/ΔV70/ΔY144)	−864.6	−11,860.4	−12,725.0	−731.7	−10,830.2	−11,561.9	−1237.9	−12,914.8	−14,152.7	−2834.2	−35,605.4	−38,439.6
Delta (ΔF157/ΔR158/L452R/T478K/D614G/P681R)	−724.0	−8939.1	−9663.1	−753.4	−9047.1	−9800.4	−687.1	−8021.5	−8708.7	−2164.5	−26,007.7	−28,172.2
Alpha(ΔH69/ΔV70/ΔY144/N501Y/A570D/D614G/P681H)	−939.2	−9293.1	−10,232.4	−950.8	−8220.7	−9171.4	−684.4	−7538.3	−8222.7	−2574.4	−25,052.1	−27,626.5
Delta_E484Q(ΔF157/ΔR158/L452R/T478K/D614G/P681R/E484Q)	−1017.0	−12,096.5	−13,113.4	−1048.5	−10,457.3	−11,505.8	−1022.2	−12,888.7	−13,910.9	−3087.6	−35,442.5	−38,530.1
Mu(T95I/Y144S/Y145N/R346K/E484K/N501Y/D614G/P681H/D950N)	−1168.3	−12,901.2	−14,069.5	−632.7	−11,080.4	−11,713.1	−1258.5	−13,583.7	−14,842.2	−3059.5	−37,565.3	−40,624.8
Mu_deltascale (ΔF157/ΔR158)	−1168.1	−12,064.9	−13,233.1	−632.3	−10,248.1	−10,880.4	−1258.0	−12,631.4	−13,889.4	−3058.4	−34,944.4	−38,002.9
Mu_alphascale (ΔH69/ΔV70/ΔY144)	−1147.2	−12,842.0	−13,989.3	−630.7	−11,062.9	−11,693.6	−1209.4	−13,442.6	−14,652.0	−2987.3	−37,347.5	−40,334.9
Omicron	−872.7	−14,757.3	−15,630.0	−570.9	−13,743.9	−14,314.8	−1081.3	−14,247.0	−15,328.3	−2524.9	−42,748.2	−45,273.1

## Data Availability

The SARS-CoV-2 whole-genome sequences described are available in GISAID (Accession IDs EPI_ISL_738142, 2361153, 2361182, 2887353, 3024839, and 8885887).

## Supplementary Materials

The following supporting information can be downloaded at: https://www.mdpi.com/article/10.3390/ijms23094956/s1.
