# Supplementary material for: Molecular Dynamics Studies on the Structural Stability Prediction of SARS-CoV-2 Variants Including Multiple Mutants
Source: Int J Mol Sci. 2022 Apr 29;23(9):4956. doi: 10.3390/ijms23094956 (PMC9106056; doi:10.3390/ijms23094956)
Supplement: Supplementary file 1 [file ijms-23-04956-s001.zip › ijms-1672443-supplementary.pdf]

Supplementary Information

Molecular Dynamics Studies on the Structural Stability Prediction of SARS-CoV-2 Variants Including Multiple Mutants

Kwang-Eun Choi [1,†], Jeong-Min Kim [2,†], Jee Eun Rhee [2], Ae Kyung Park [2], Eun-Jin Kim [2], Cheon Kwon Yoo[3], and Nam Sook Kang [1,*]

[1] Graduate School of New Drug Discovery and Development, Chungnam National University, 99 Daehak-ro, Yuseong-gu, Daejeon 34134, Korea; hwendiv@naver.com
[2] Division of Emerging Infectious Diseases, Bureau of Infectious Disease Diagnosis Control, Korea Disease Control and Prevention Agency, 187 Osongsaengmyeong 2-ro, Osong-eup, Heungdeok-gu,
Cheongju-si 28159, Korea; jmkim97@korea.kr (J.-M.K.); jerhee001@korea.kr (J.R.); parkak1003@korea.kr (A.K.P.); ekim@korea.kr (E.-J.K.)
[3] Bureau of Infectious Disease Diagnosis Control, Korea Disease Control and Prevention Agency, 187 Osongsaengmyeong 2-ro, Osong-eup, Heungdeok-gu, Cheongju-si 28159, Korea; ckyoo@korea.kr (C.K.Y.)

* Correspondence: nskang@cnu.ac.kr; Tel.: +82-42-821-8626
† These authors contributed equally to this work.

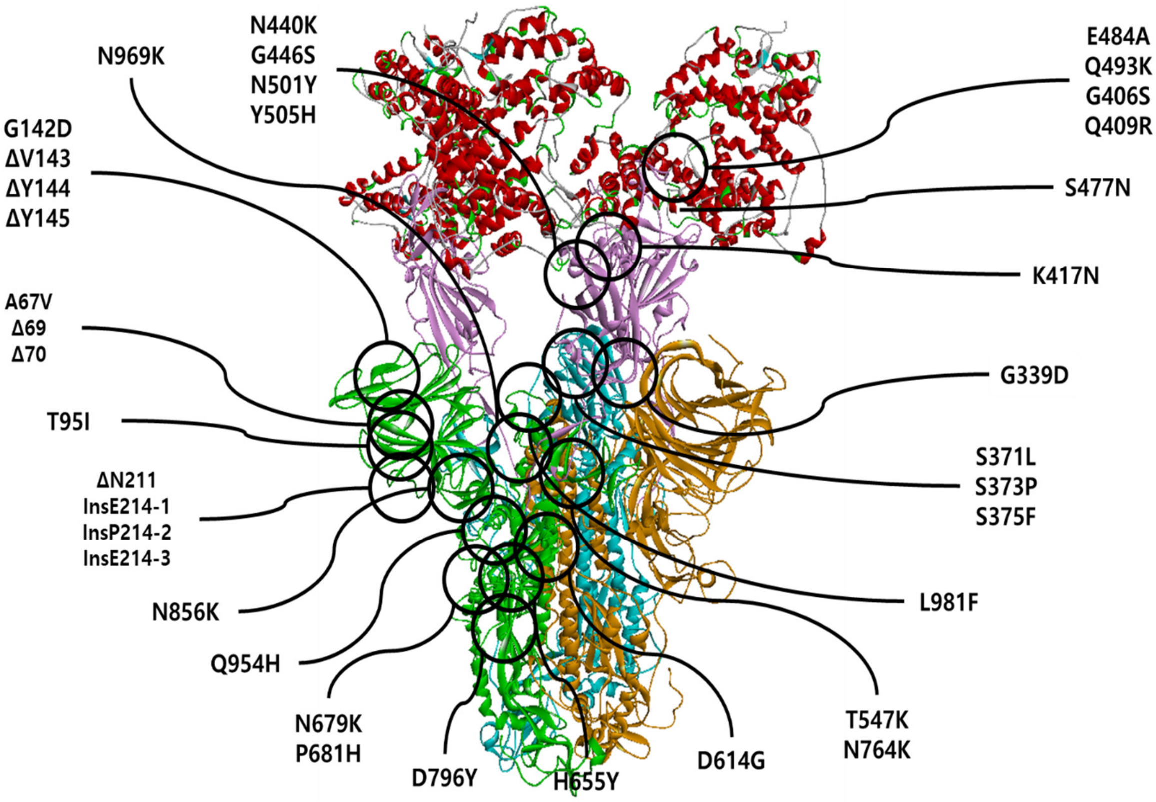

Figure S1. Mutation sites of S protein for omicron variant. Trimeric RBDs in each chain are indicated in pink and ACE2 in red. Yellow, blue, and green colors indicate other domains except for RBD in each chain of S protein.

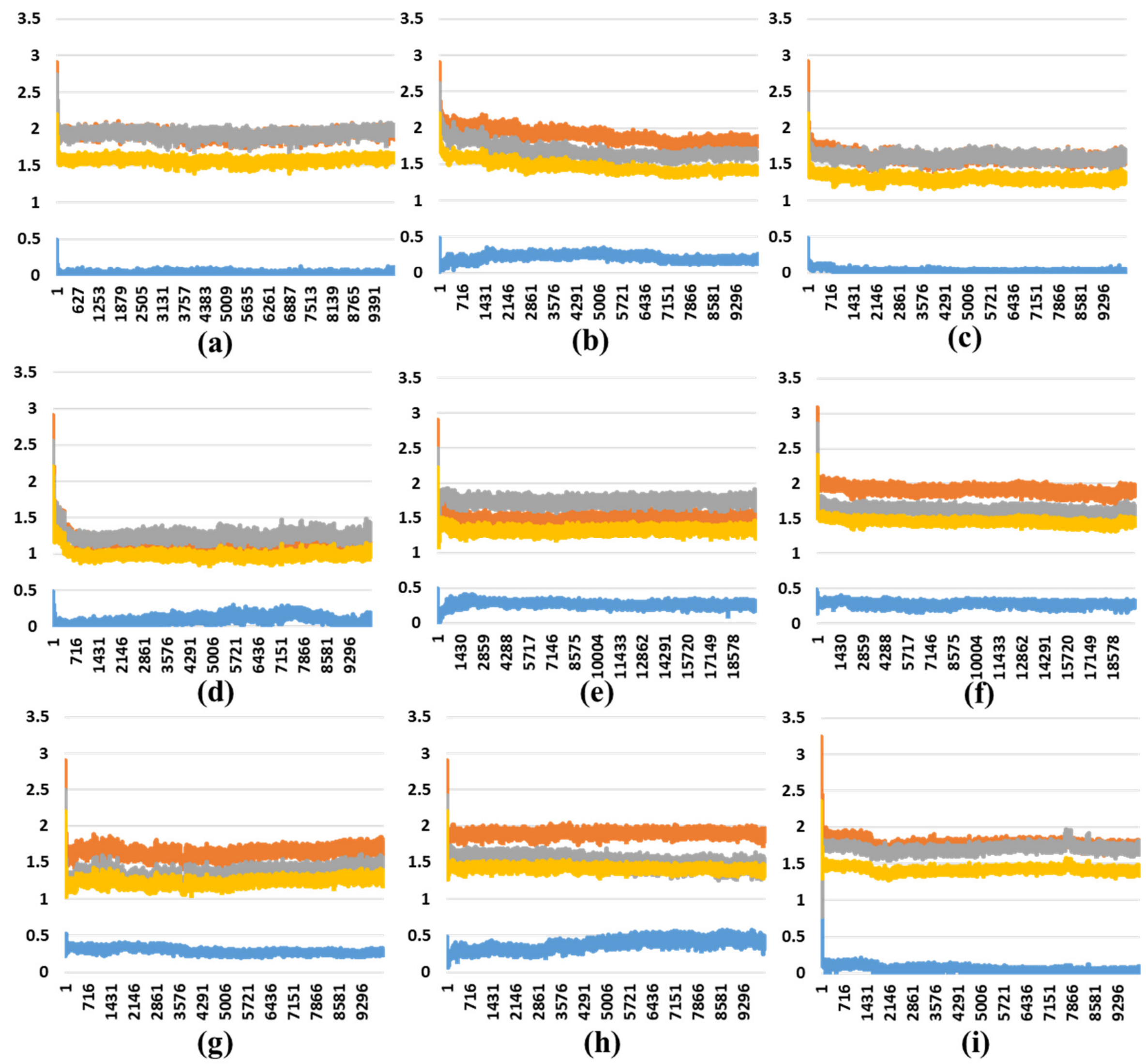

Figure S2. The standard deviation for distance between V503 residues in each chain depending on MD trajectories in the one-open-complex form (7A94). (**a**) Wild type and (**b**)-(**i**) variants. (**b**) double (**c**) triple (**d**) B.1620 (**e**) delta (**f**) alpha (**g**) delta_E484Q (**h**) mu and (**i**) omicron. The X axis denotes trajectory (ps) and the Y axis denotes standard deviation for distance (nm). Blue color indicates the standard deviation between AB and BC chain, orange indicates that between AB and AC chain, gray indicates that between AC and BC chain, and yellow indicates that among A,B, and C chain.

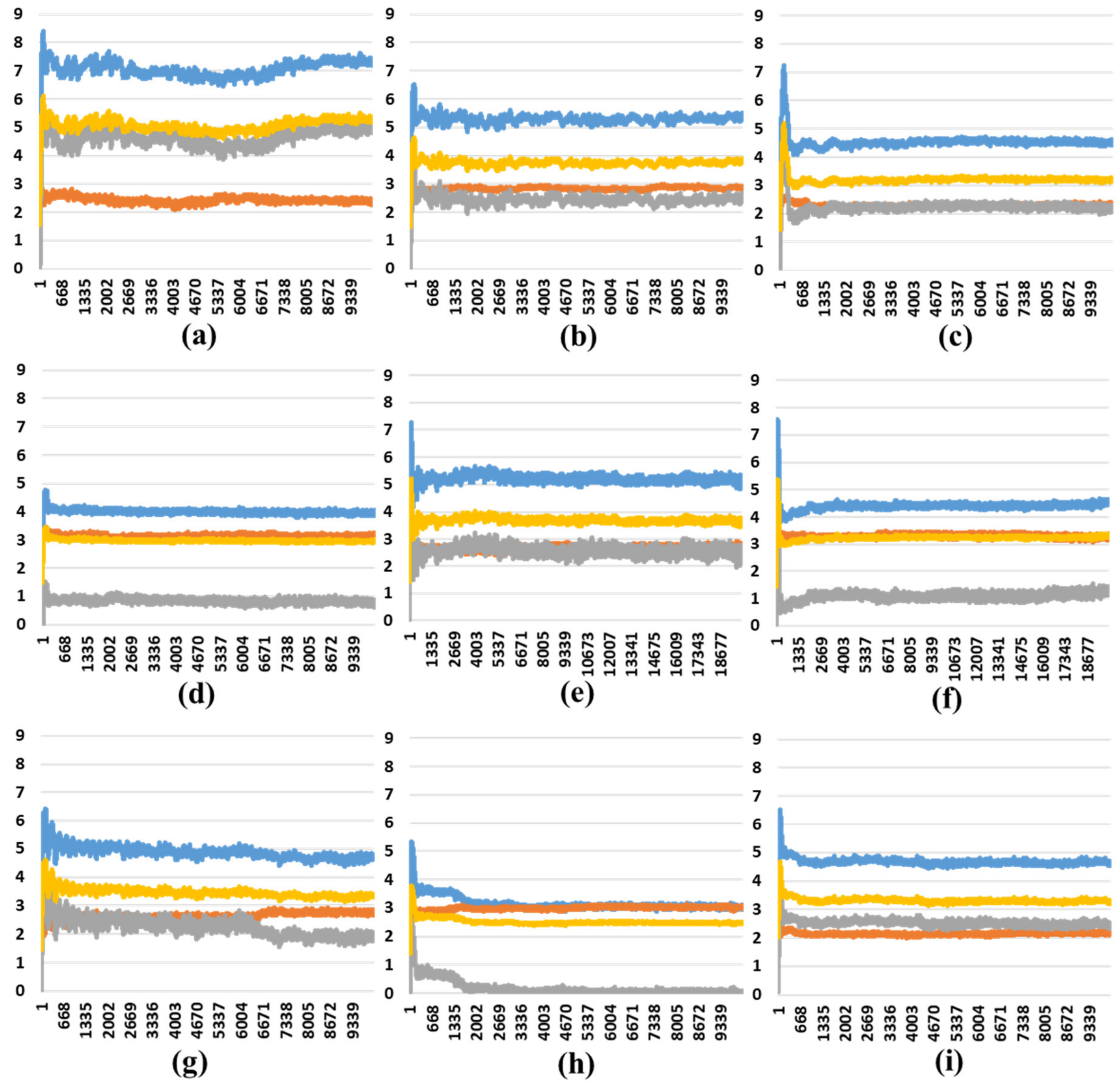

Figure S3. The standard deviation for distance between V503 residues in each chain depending on MD trajectories in the two-open-complex form (7A97) (**a**) Wild type and (**b**)-(**i**) variants. (**b**) double (**c**) triple (**d**) B.1620 (**e**) delta (**f**) alpha (**g**) delta_E484Q (**h**) mu and (**i**) omicron. The X axis denotes trajectory (ps) and the Y axis denotes standard deviation for distance (nm). Blue color indicates the standard deviation between AB and BC chain, orange indicates that between AB and AC chain, gray indicates that between AC and BC chain, and yellow indicates that among A,B, and C chain.

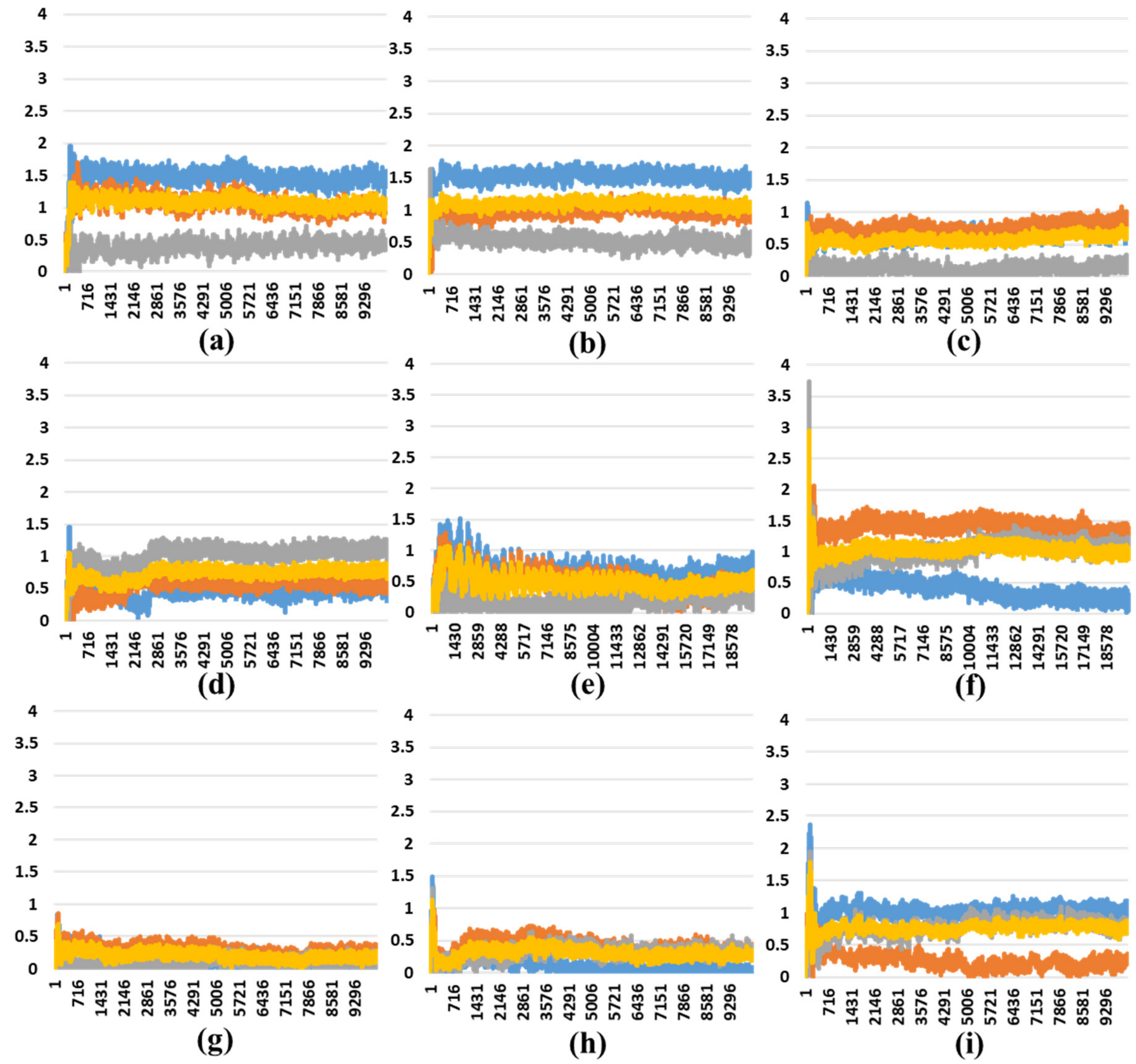

Figure S4. The standard deviation for distance between V503 residues in each chain depending on MD trajectories in the three-open-complex form (7A98). (**a**) Wild type and (**b**)-(**i**) variants. (**b**) double (**c**) triple (**d**) B.1620 (**e**) delta (**f**) alpha (**g**) delta_E484Q (**h**) mu and (**i**) omicron. The X axis denotes trajectory (ps) and the Y axis denotes standard deviation for distance (nm). Blue color indicates the standard deviation between AB and BC chain, orange indicates that between AB and AC chain, gray indicates that between AC and BC chain, and yellow indicates that among A,B, and C chain.

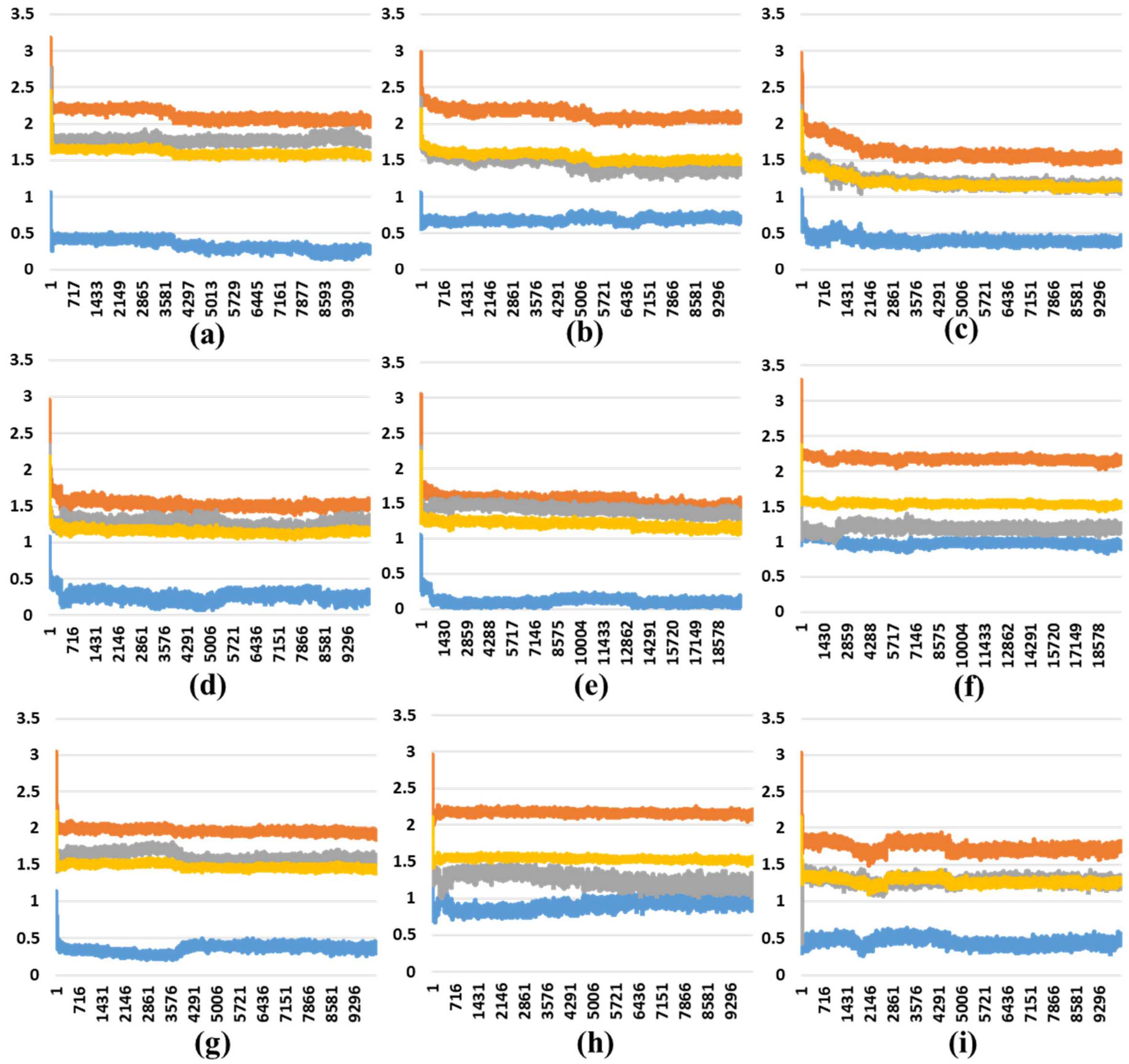

Figure S5. The standard deviation for distance between N501 residues in each chain depending on MD trajectories in the one-open-complex form (7A94). (**a**) Wild type and (**b**)-(**i**) variants. (**b**) double (**c**) triple (**d**) B.1620 (**e**) delta (**f**) alpha (**g**) delta_E484Q (**h**) mu and (**i**) omicron. The X axis denotes trajectory (ps) and the Y axis denotes standard deviation for distance (nm). Blue color indicates the standard deviation between AB and BC chain, orange indicates that between AB and AC chain, gray indicates that between AC and BC chain, and yellow indicates that among A,B, and C chain.

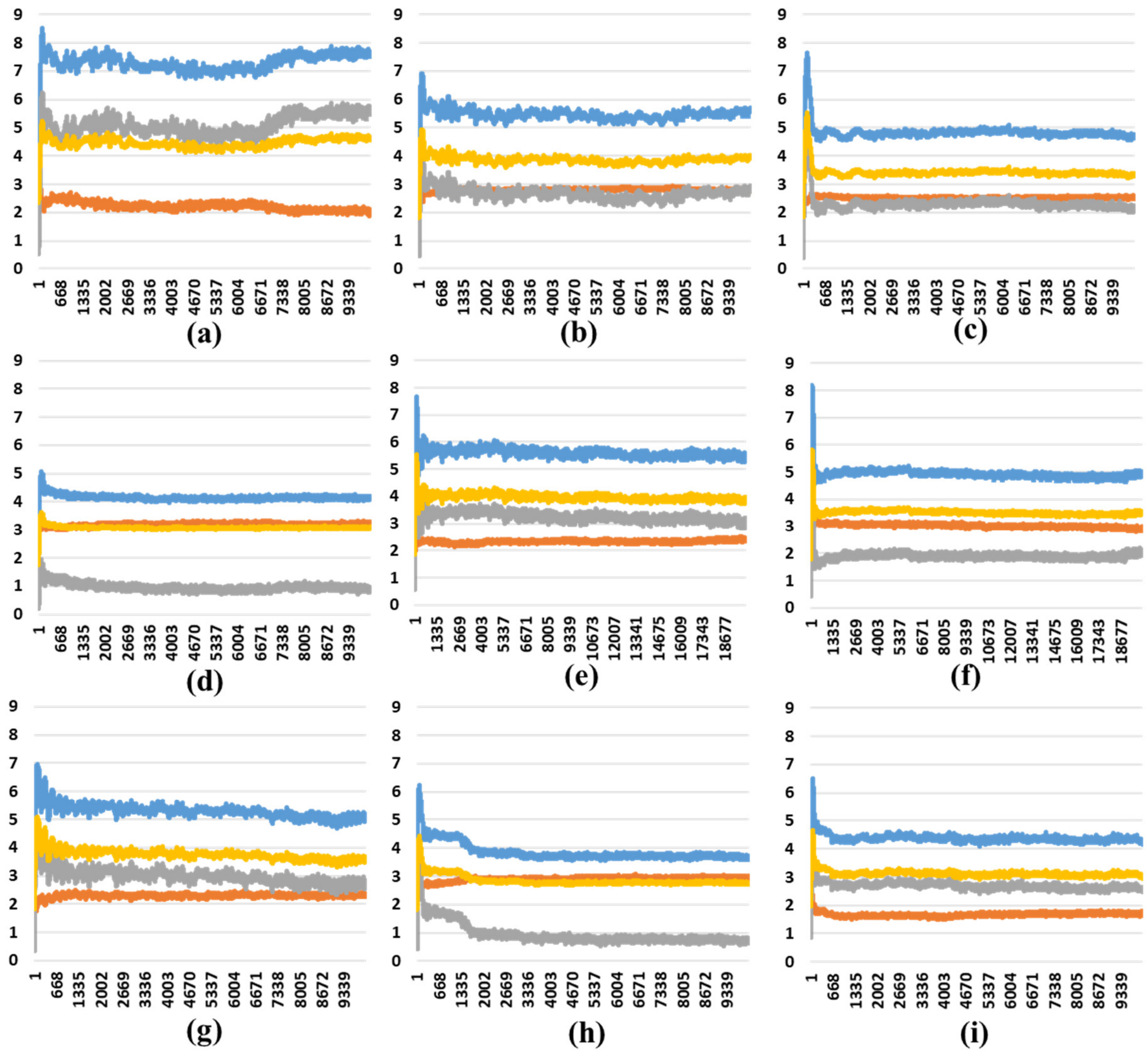

Figure S6. The standard deviation for distance between N501 residues in each chain depending on MD trajectories in the two-open-complex form (7A97). (**a**) Wild type and (**b**)-(**i**) variants. (**b**) double (**c**) triple (**d**) B.1620 (**e**) delta (**f**) alpha (**g**) delta_E484Q (**h**) mu and (**i**) omicron. The X axis denotes trajectory (ps) and the Y axis denotes standard deviation for distance (nm). Blue color indicates the standard deviation between AB and BC chain, orange indicates that between AB and AC chain, gray indicates that between AC and BC chain, and yellow indicates that among A,B, and C chain.

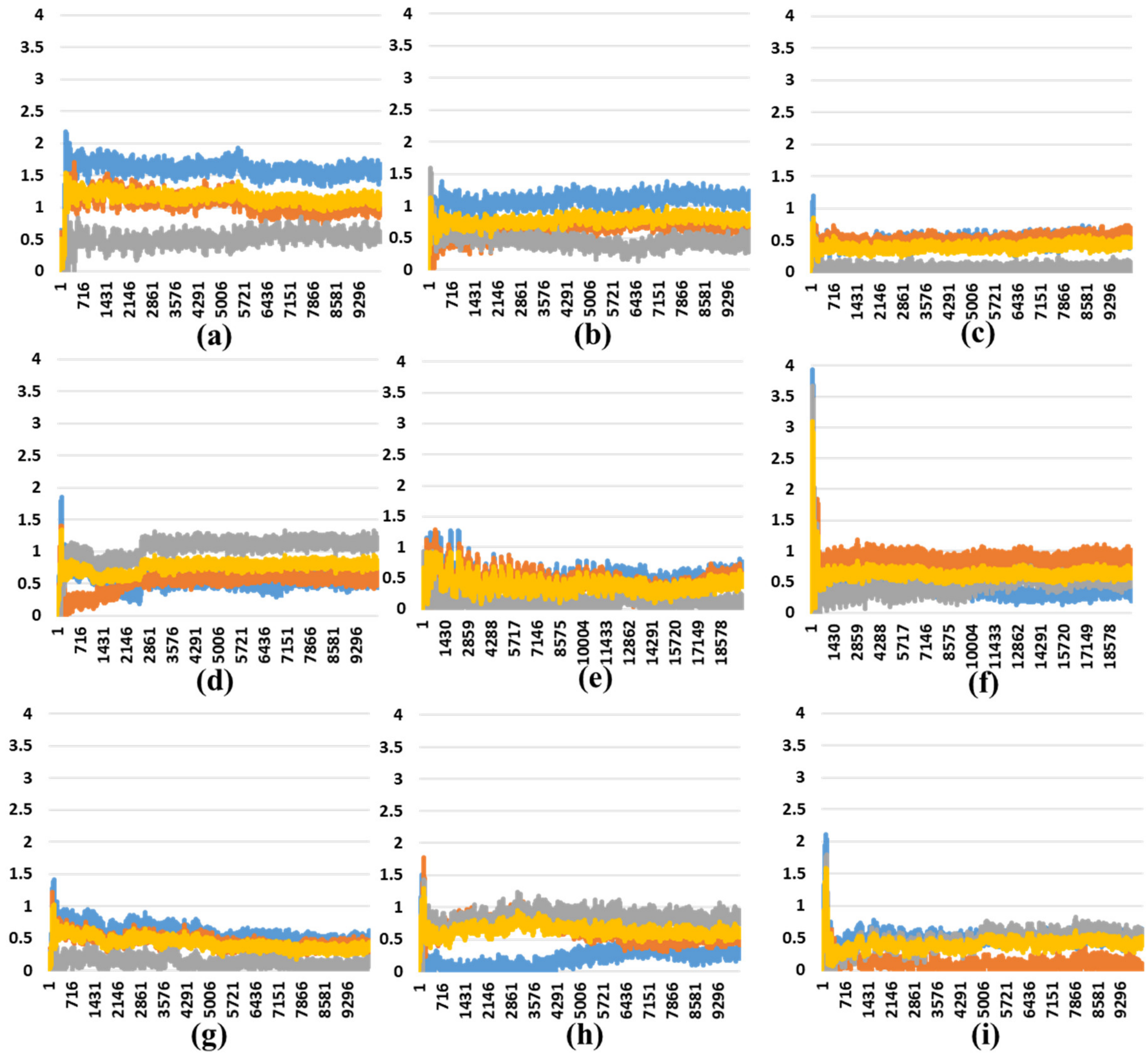

Figure S7. The standard deviation for distance between N501 residues in each chain depending on MD trajectories in the three-open-complex form (7A98). (**a**) Wild type and (**b**)-(**i**) variants. (**b**) double (**c**) triple (**d**) B.1620 (**e**) delta (**f**) alpha (**g**) delta_E484Q (**h**) mu and (**i**) omicron. The X axis denotes trajectory (ps) and the Y axis denotes standard deviation for distance (nm). Blue color indicates the standard deviation between AB and BC chain, orange indicates that between AB and AC chain, gray indicates that between AC and BC chain, and yellow indicates that among A,B, and C chain.

Table S1. Summary of distance and SD between N501 residues (nm). The values were measured in the final trajectory (10 ns).

PDB	Variant Type	A-B	A-C	B-C	S.D. (A-B) & (A-C)	S.D. (A-B) & (B-C)	S.D. (A-C) & (B-C)	S.D. (A-B-C)
7A94 (one-open complex form)	**Wild**	3.33	2.91	0.43	0.30	2.05	1.75	1.57
	**D614G/E484K**	3.55	2.54	0.64	0.72	2.06	1.35	1.48
	**D614G/E484K/N440K**	3.10	2.57	0.85	0.38	1.59	1.21	1.18
	**B.1.620**	2.90	2.60	0.77	0.21	1.51	1.51	1.15
	**Delta**	2.63	2.51	0.56	0.09	1.47	1.38	1.17
	**England**	3.63	2.27	0.60	0.96	2.14	1.18	1.52
	**Delta (+E484Q)**	3.33	2.77	0.62	0.39	1.92	1.52	1.43
	**Mu**	3.73	2.41	0.80	0.93	2.07	1.14	1.47
	**Omicron**	3.57	2.85	1.16	0.51	1.71	1.19	1.24
7A97 (two-open complex form)	**Wild**	13.93	3.10	11.08	7.66	2.02	5.64	4.66
	**D614G/E484K**	11.26	3.31	7.33	5.63	2.78	2.85	3.98
	**D614G/E484K/N440K**	9.97	3.37	6.32	4.67	2.58	2.08	3.31
	**B.1.620**	10.13	4.27	5.55	4.14	3.24	3.24	3.08
	**Delta**	11.29	3.49	7.87	5.52	2.42	3.09	3.91
	**England**	10.65	3.69	6.60	4.92	2.87	2.05	3.50
	**Delta (+E484Q)**	10.56	3.47	7.21	5.02	2.37	2.65	3.55
	**Mu**	8.59	3.37	4.36	3.69	2.99	0.70	2.77
	**Omicron**	8.99	3.02	6.57	4.22	1.71	2.50	3.00

7A98 (three-open complex form)	**Wild**	8.04	10.29	9.56	1.59	1.07	0.52	1.15
	**D614G/E484K**	8.74	10.20	9.60	1.03	0.60	0.43	0.73
	**D614G/E484K/N440K**	8.62	9.44	9.53	0.58	0.64	0.06	0.50
	**B.1.620**	9.75	9.03	10.62	0.51	0.62	0.62	0.80
	**Delta**	8.88	9.52	9.68	0.46	0.57	0.11	0.43
	**England**	9.32	9.86	10.62	0.38	0.92	0.54	0.65
	**Delta (+E484Q)**	10.04	9.39	9.55	0.46	0.35	0.11	0.34
	**Mu**	10.04	10.62	9.36	0.41	0.48	0.90	0.63
	**Omicron**	9.71	8.98	9.69	0.52	0.02	0.50	0.42

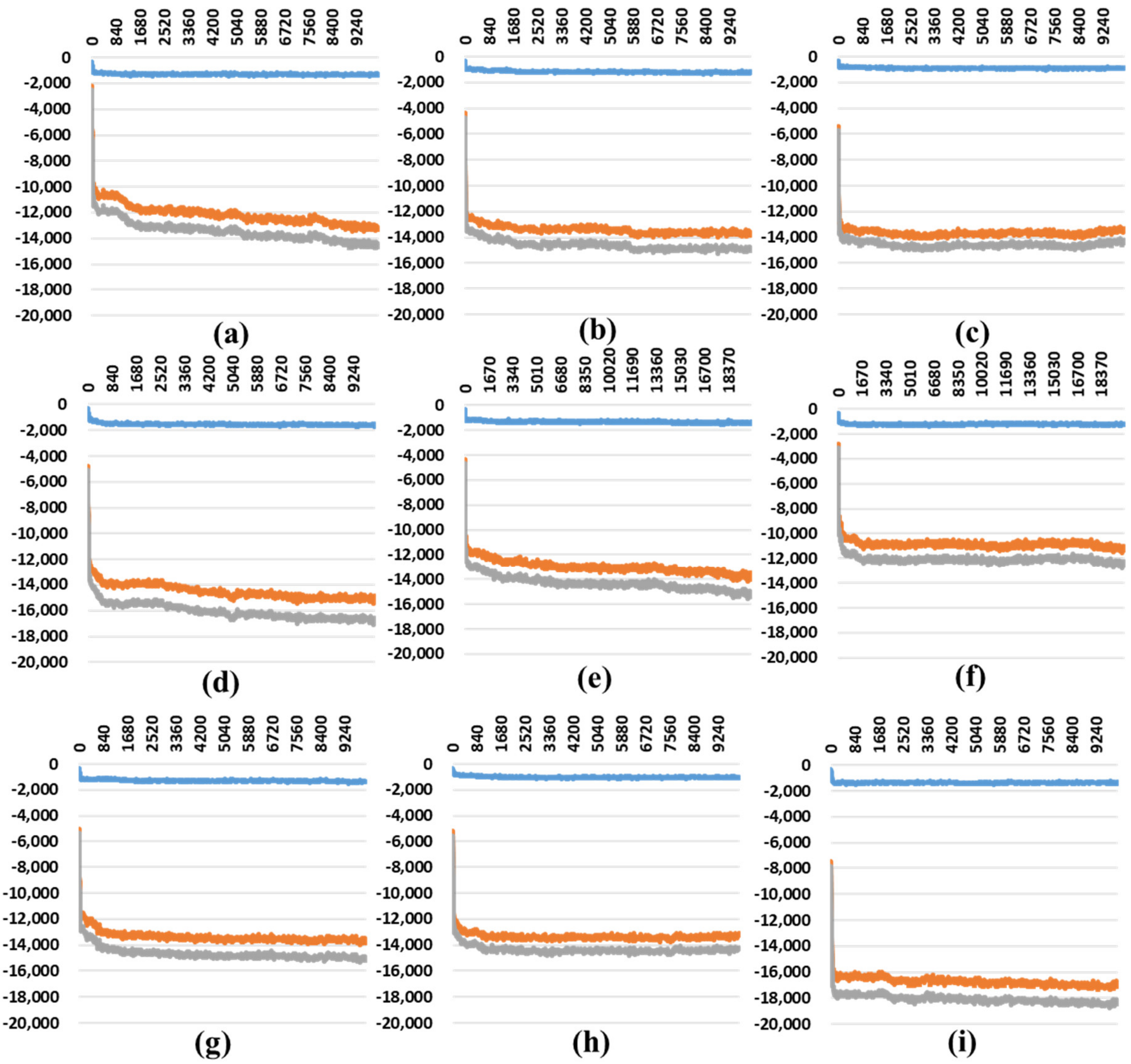

(1) A chain (S protein) – D chain (ACE2)

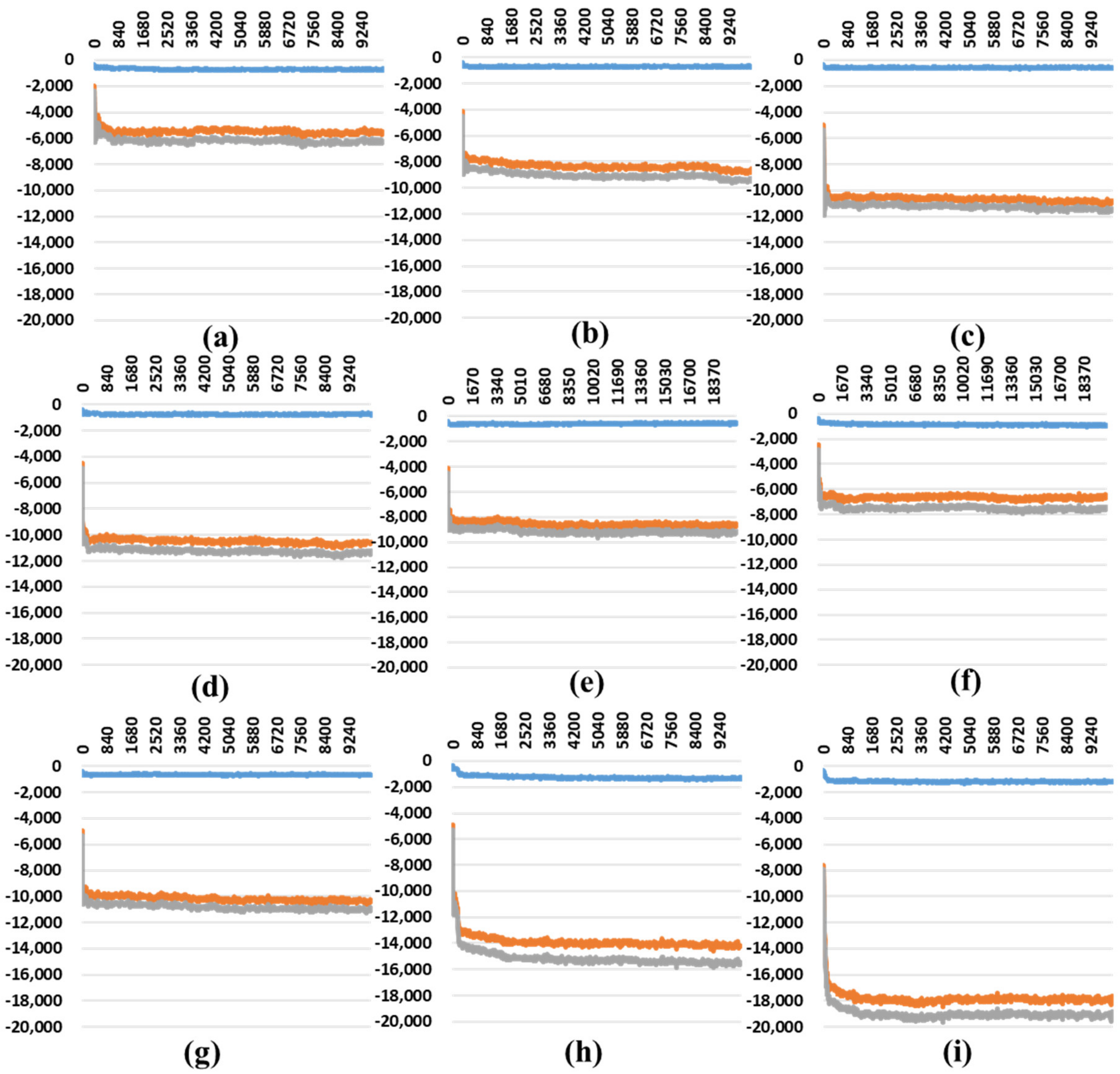

(2) B chain (S protein) – E chain (ACE2)

Figure S8. MM/PBSA results for A–D (1) and B–E (2) depending on the MD trajectories in the two-open-complex form (7A97). (**a**) Wild type and (**b**)-(**i**) variants. (**b**) double (**c**) triple (**d**) B.1620 (**e**) delta (**f**) alpha (**g**) delta_E484Q (**h**) mu and (**i**) omicron. The X axis denotes trajectory (ps), and the Y axis denotes the MM/PBSA energy value (kJ/mol). Blue color indicates van der Waals energy between S protein and ACE2 , orange color indicates electrostatic energy, and gray color indicates total energy.

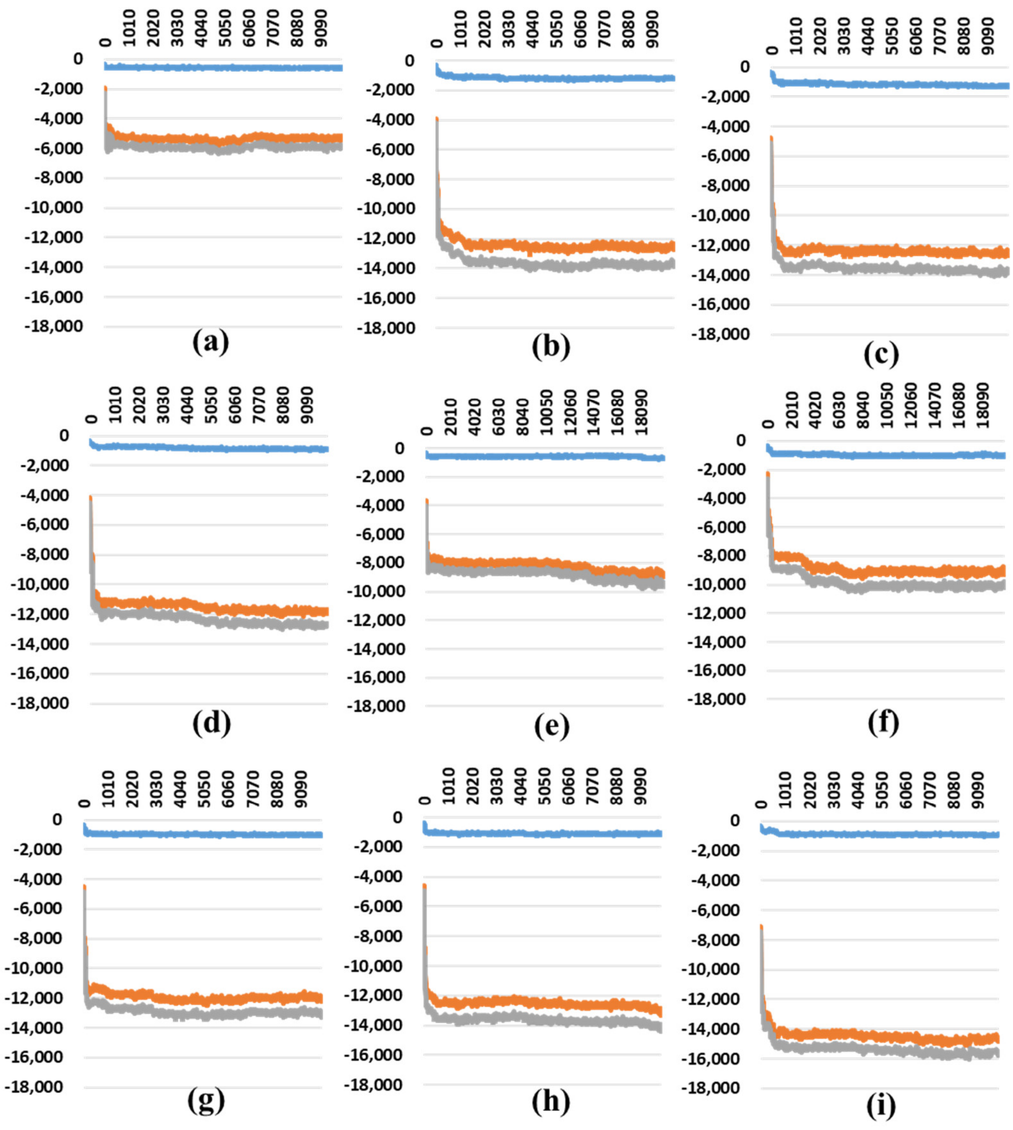

(1) A chain (S protein) – D chain (ACE2)

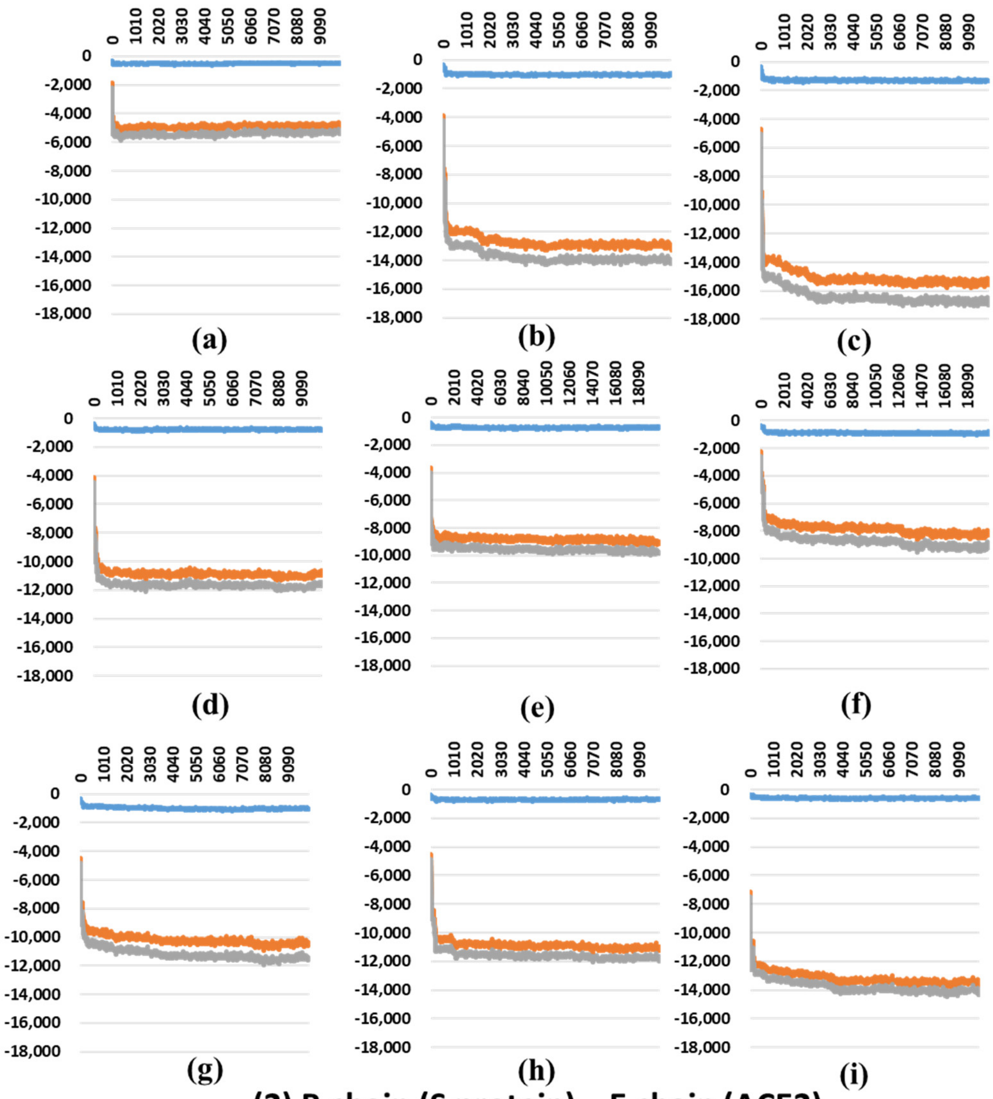

(2) B chain (S protein) – E chain (ACE2)

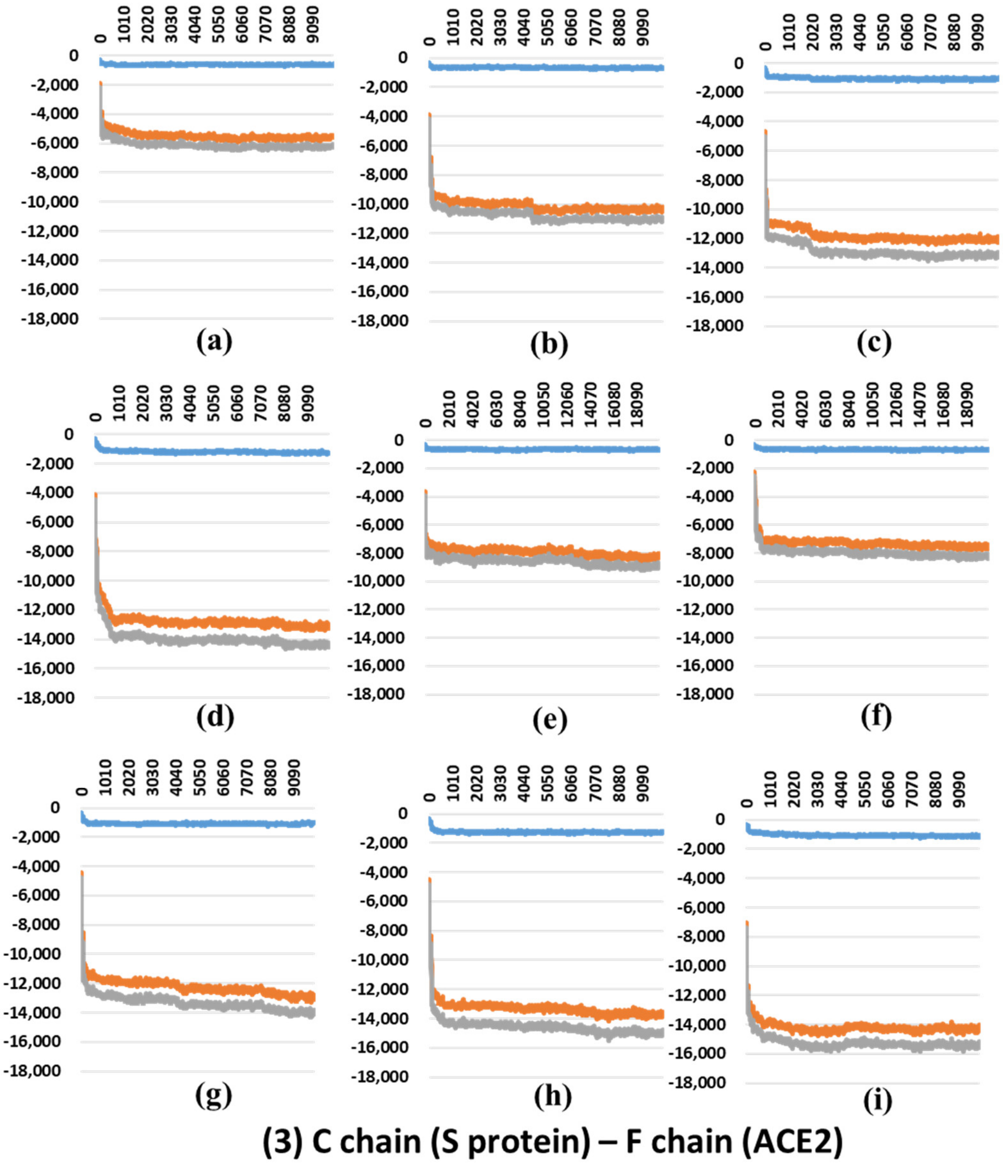

Figure S9. MM/PBSA for A–D (1), B–E (2), and C–F (3) depending on the MD trajectories in the three-open-complex form (7A98). (**a**) Wild type and (**b**)-(**i**) variants. (**b**) double (**c**) triple (**d**) B.1620 (**e**) delta (**f**) alpha (**g**) delta_E484Q (**h**) mu and (**i**) omicron. The X axis denotes trajectory (ps), and the Y axis denotes MM/PBSA energy value (kJ/mol). Blue color indicates van der Waals energy between S protein and ACE2, orange color indicates electrostatic energy.
